# Analysis of Factors Causing Skin Damage in the Application of Peripherally Inserted Central Catheter in Cancer Patients

**DOI:** 10.1155/2021/6628473

**Published:** 2021-03-16

**Authors:** Luan Tian, Xinxin Yin, Yuxin Zhu, Xin Zhang, Congcong Zhang

**Affiliations:** ^1^Department of Digestive, Shijiazhuang People's Hospital, Shijiazhuang, China; ^2^Department of Central Venous Catheter Clinic, Shijiazhuang People's Hospital, Shijiazhuang, China; ^3^Shijiazhuang People's Hospital, Jianhua South Street, Yuhua District, Shijiazhuang City 050000, China

## Abstract

**Objective:**

To investigate the related factors of skin damage caused by peripherally inserted central catheter (PICC) in cancer patients.

**Methods:**

It was a retrospective analysis of 202 cancer patients admitted to our hospital from February 2014 to July 2019. 50 cases of PICC-related skin damage and 152 cases of non-skin damage were studied. In addition, multivariate logistic regression analysis was used to determine independent risk factors for PICC-related skin damage, including cancer patients with catheter-related skin damage and patients without skin damage.

**Results:**

50 patients with PICC skin damage (19 males and 31 females) and 152 patients without skin damage (62 males and 90 females) were retrospectively analyzed. The skin damage rate was 24.8%. The analysis of variance results showed that many factors are related to PICC catheter-related skin damage, including hormones (*χ*^2^/*Z* = 4.468, *P* < 0.05), body mass index (BMI) (*χ*^2^/*Z* = −2.443, *P* < 0.05), days with tube (*χ*^2^ = 26.230, *P* < 0.05), chemotherapy cycle (*χ*^2^/*Z* = 25.638, *P* < 0.05), and self-care ability (*χ*^2^/*Z* = −1.968, *P* < 0.05). Logistic regression analysis showed that hormones (odds ratio (OR) = 3.896, *P*=0.045), BMI (OR = 1.129, *P*=0.017), days with tube (OR = 0.419, *P*=0.013), and chemotherapy cycle (OR = 3.302, *P*=0.028) are independent factors affecting PICC-related skin damage.

**Conclusion:**

The independent influencing factors of skin damage during PICC catheterization are hormones, BMI, number of days with tube, and chemotherapy cycle.

## 1. Introduction

Peripherally inserted central catheters (PICCs) are vascular access devices [1], which have the advantages of simple operation, safety, and long catheter indwelling time [2]. In recent years, PICCs have been more and more widely used in the course of chemotherapy for cancer patients. How to quickly discover complications and risk factors has become a key factor in ensuring the safety of patients' lives. Although PICC provides a lot of convenience to patients and medical staff [3], the occurrence of complications will also weaken these advantages. Skin damage is a common complication associated with PICC [4]. Repeated application and removal of dressings at the PICC site often lead to skin damage [5]. Long-term indwelling PICC catheters in some patients are prone to iatrogenic skin damage, which increases patient suffering and even affects clinical treatment, which is not conducive to recovery [6]. According to clinical observation, the catheter needs to be fixed with a transparent adhesive film based on the successful PICC catheterization. The length of the catheterization time, the patient's physique, the chemotherapy cycle, the use of hormones, and other factors can cause skin allergies, eczema, and other problems to general patients [7, 8]. In mild cases, the skin irritation affects the daily life of the patient, and in the worst case, it may aggravate the complications such as early extubation and skin redamage, affecting the life of PICC and increasing the economic burden of patients [9].

Therefore, analyzing the risk factors of PICC-related skin damage has important clinical value. In this study, logistic regression analysis was used to investigate the related factors of PICC-related skin damage in cancer patients. When formulating a targeted treatment plan, clinicians can consider related risk factors to provide a basis for reducing PICC catheter-related skin damage.

## 2. Patients and Methods

### 2.1. Patients

We retrospectively collected the clinical data of 202 cancer patients from February 2018 to July 2019 and included a total of 50 cases in PICC-related skin damage group and 152 cases in non-skin damage group. Among them, there were 81 males and 121 females. Other information, including age, diabetes, bed rest, history of allergies, skin shape, chemotherapy drug use, hormone use (dexamethasone), history of radiotherapy, number of days with tube, specialist nurse, chemotherapy cycle, body mass index (BMI), and self-care ability are shown in Table 1.

### 2.2. Inclusion and Exclusion Criteria

The inclusion criteria are (1) patients who need PICC according to their condition and treatment; (2) patients with PICC-related skin damage; (3) patients with complete clinical data. Exclusion criteria are as follows: (1) patients' diseases are not accompanied by skin diseases or skin damage.

### 2.3. Statistical Analysis

SPSS 25.0 statistical software was used for data analysis. Count data was expressed by frequency and percentage (%), and the comparison between groups was performed by *χ*^2^ test. Nonnormally distributed measurement data were represented by median and quartile [M (P_25_, P_75_)], and rank sum test was used for comparison between groups. The difference is statistically significant when *P* < 0.05.

## 3. Results

### 3.1. Univariate Analysis of Skin Damage

A total of 202 patients with PICC-related skin damage were included, including 81 males and 121 females. The incidence of PICC-related skin damage was 24.75%, of which men accounted for 23.5% and women accounted for 25.6%. The analysis results show that, whether or not to use hormones, the number of days with tube, BMI, self-care ability, and the chemotherapy cycle are statistically significant in comparing whether there is skin damage during PICC catheterization (*P* < 0.05); see Table 1.

### 3.2. Binary Logistic Regression Analysis

Taking whether the patient had skin damage as the dependent variable, and whether using hormones, the number of days with tube, BMI, self-care ability, and chemotherapy cycle as independent variables, a binary logistic regression analysis was performed. The variable assignment is shown in Table 2. The results show that hormone use, number of days with tube, BMI, and chemotherapy cycle are the influencing factors for skin damage during PICC catheterization; see Table 3. The results show that hormone use is most closely related to PICC-related skin damage, followed by chemotherapy cycles, BMI, and days with tube.

## 4. Discussion

PICC is a catheter inserted into the superior vena cava through a peripheral vein. The PICC can be used to infuse drugs into the central vein with large blood flow and fast flow rate to prevent patients from blood vessels damage due to long-term infusion or infusion of hypertonic or irritating drugs and reduce the pain caused by repeated punctures. Therefore, it is often used in patients who need chemotherapy. Although PICC catheter types, indwelling methods, fixation methods, and medical dressings have been continuously improved, catheter-related skin damage still cannot be completely avoided. This is an important issue that requires clinical medical staff to pay close attention [10, 11]. If the skin damage caused by PICC is not treated promptly and appropriately, it will cause more serious problems, such as skin diseases, various infections, and tissue damage, which will affect the treatment effect and quality of life of the patient and increase the patient's medical expenses [12, 13].

Studies have shown that the incidence of PICC-related medical adhesive-related skin damage in cancer patients is as high as 33.99% [7, 14, 15]. Compared with the general population, PICC-related skin damage has a higher incidence in this population [16]. Over the years, many scholars have carried out correlation analysis of many risk factors for PICC skin damage caused by medical adhesive, including the material of medical tape, the type of adhesive, and improper operation techniques. However, there are few literatures on the regression analysis of the correlation between the type of chemotherapy drugs, chemotherapy cycle, hormone use, catheter indwelling time, self-care ability, and PICC-related skin damage.

A total of 14 related indicators are included in this study. Univariate analysis shows that there are significant differences in hormone use, days with tube, BMI, self-care ability, and chemotherapy cycle between patients with skin damage and those without skin damage (*P* < 0.05). It shows that these five factors may be related to PICC-related skin damage. In this study, logistic regression method is used to analyze related factors affecting PICC-related skin damage. The logistic multivariate analysis is used to correct the confounding factors, avoiding the one-sidedness of the univariate analysis results. The results show that PICC-related skin damage is affected by a variety of factors. These factors are related not only to the level of operators and catheter care staff, but also to the patient's personal physique and medical history. In addition, they are also closely related to factors such as hormone use, chemotherapy cycles, and patient BMI. Hormone is an independent factor influencing whether there is skin damage during PICC catheterization (*P*=0.045). Patients who use hormones are 3.896 times more likely to have skin damage than those who do not use hormones, which means that the use of hormones is a risk factor for skin damage during PICC catheterization. This is related to the repeated use of hormones to fight chemosensitivity in cancer patients, which may cause the skin's dermis to shrink and blood vessels to dilate to form hormone-dependent dermatitis. BMI is an independent factor influencing whether there is skin damage during PICC indwelling process (*P*=0.011 < 0.05), and the influence coefficient is 0.122>0, which means that the higher the patient's BMI, the higher the possibility of damage during catheter indwelling. Therefore, BMI is a risk factor for skin damage during PICC catheterization. Patients with BMI increased by 1 unit are 1.129 times more likely to have skin damage than patients with BMI lower by 1 unit. Patients with increased BMI are obese and sweat easily, and the risk of skin rashes is greater for the use of adhesive base dressings. The number of days with tube is an independent factor influencing whether there is skin damage during the PICC indwelling process (*P*=0.013 < 0.05), and the influence coefficient is −0.870 > 0. Each time the number of days with tube increases by one grade, the probability of skin damage is 0.419 times that of patients with a lower grade. The statistical results of this study show that the longer the patient has been with the tube, the lower the possibility of skin damage is. The length of days with tube is not the main factor leading to skin damage during the PICC indwelling process. There is no positive correlation between the number of days with tube and the skin damage of the patient. The possible reason is that the skin damage is getting heavier after treatment, and the tube is extubated in advance, or the patient with poor treatment effect requires extubation. According to clinical observation, some patients continued to retain the catheter after treatment and recovered, and no skin damage occurred. Studies have found that the indwelling time of the catheter can significantly affect the occurrence of PICC-related infections [17, 18], but the effect on skin damage is rarely reported. The chemotherapy cycle is an independent factor influencing whether there is skin damage during PICC catheterization (*P*=0.028 < 0.05). Patients with a chemotherapy cycle ≥ stage IV are 1.109 times more likely to have skin damage than patients with stage ＜IV, which means chemotherapy cycle is a risk factor for skin damage during PICC catheterization. Most chemotherapy drugs have adverse reactions that cause dermatitis, and the decline in the patient's resistance after chemotherapy is also one of the factors that lead to skin damage. Although there are many reports in the literature that the BMI of cancer patients is closely related to the risk of PICC infection [19, 20], so far, our study is the first to find a link between hormone use, BMI, number of days with tube, chemotherapy cycle, and PICC-related skin damage. Few other studies have explored the correlation between patients' BMI, number of days with tube, chemotherapy cycle, and PICC-related skin damage.

## 5. Conclusion

This study analyzed the factors of PICC's skin damage to cancer patients. The use of hormones, BMI, chemotherapy cycle, and the number of days with tube are independent factors that affect the skin damage of cancer patients during PICC catheterization. After analysis, we found that, for cancer patients with repeated use of hormones, increased BMI, and chemotherapy, we need to assess the skin condition during catheter indwelling in advance. Once a rash occurs, use gauze dressing as soon as possible and avoid contact with adhesive based dressings, so as to reduce the incidence of catheter-related skin damage in cancer patients. Due to limited medical records, certain deviations are inevitable. There may be other influencing factors for PICC-related skin damage in cancer patients. Therefore, a comprehensive analysis of individual factors in cancer patients may have more clinical reference significance for reducing complications such as skin damage and exerting the application value of PICC. However, the main limitation of this study is that the data source is retrospective data, and the sample size is small. It is suggested that prospective cohort study should be carried out in the follow-up study to further clarify the risk factors of skin injury.

## Figures and Tables

**Table 1:** Comparison of skin injury in PICC catheterization with different data (*n* = 202).

Item	Skin injury of PICC catheter		Incidence of skin injury (%)	*χ* ^2^/*Z*	*P* value
	No	Yes			
Sex (case)					
Male	62	19	23.5	0.122	0.727
Female	90	31	25.6		

Diabetes					
Yes	37	10	21.3	0.397	0.528
No	115	40	25.8		

Stay in bed					
Yes	14	3	17.7	0.503	0.478
No	138	47	25.4		

Allergy history					
Yes	8	5	38.5	1.402	0.236
No	144	45	23.8		

Skin characteristics					
Oiliness	15	8	34.8	1.430	0.489
Neutral	122	37	23.3		
Dryness	15	5	25.0		

Category of chemotherapeutic drugs					
Vesicant	27	8	22.9	2.839	0.242
Non bullous	125	42	25.1		

Whether or not use of hormones					
Yes	124	47	27.5	4.468	0.035
No	28	3	9.7		

History of radiotherapy					
Yes	32	15	31.9	1.687	0.194
No	120	35	22.6		

Days with tube					
≤90	25	25	50.0	26.230	<0.001
91–180	84	22	20.8		
≥181	43	3	6.5		

Specialist nurse					
Yes	101	38	27.3	1.600	0.206
No	51	12	19.1		

Chemotherapy cycle					
<IV stage	19	23	54.8	25.638	<0.001
≥IV stage	133	27	16.9		

Age (years) ^#^	[61.0 (53.0, 67.0)]	[56.5 (40.0, 65.2)]		−1.792	0.073
BMI ^#^	[23.6 (21.5, 27.7)]	[24.5 (22.8, 27.9)]		−2.443	0.015
Self care ability (score) ^#^	[90.0 (85.0, 100.0)]	[100.0 (88.8, 100.0)]		−1.968	0.049

^#^The data are nonnormal distribution after normal test. Vesicant: Drugs can damage the vascular wall and cause irreversible inflammatory changes. Non-bullous: Drugs can damage the blood vessel wall and cause reversible inflammatory changes.

**Table 2:** Binary logistic regression analysis variable assignment of skin injury during PICC catheterization.

Variable	Assignment
Whether or not skin damage occurs	No = 0, yes = 1
Whether or not use of hormones	No = 0, yes = 1
Chemotherapy cycle	<IV stage = 0, ≥IV stage = 1
Tube time	Original value entry
BMI	Original value entry
Self care ability	Original value entry

**Table 3:** Results of binary logistic regression analysis on whether skin injury occurred during PICC catheterization.

	Regression coefficient	Standard error	Wald value	*P*	OR value	OR value 95% confidence interval	
						Lower limit	Upper limit
Constant	−5.780	2.274	6.463	0.011	0.003	—	—
Whether or not use of hormones (yes)	1.360	0.679	4.012	0.045	3.896	1.030	14.743
BMI	0.122	0.051	5.651	0.017	1.129	1.022	1.249
Days with tube	−0.870	0.352	6.126	0.013	0.419	0.210	0.834
Chemotherapy cycle (≥IV stage)	1.109	0.504	4.848	0.028	3.032	1.130	8.139

Only the independent variables with *P* < 0.05 are shown. The accuracy of these variables in predicting skin injury was 81.7%. ‘OR Value' (odd ratio value) is the ratio index to measure the effect of risk factors. Wald is a chi square value and it is equal to B divided by the square of its standard error (S.E.).

## Data Availability

The data used are available from the corresponding author upon reasonable request.
